# Appropriate Scaffold Selection for CNS Tissue Engineering

**Published:** 2020

**Authors:** Akram Shafiee, Hanie Ahmadi, Behnaz Taheri, Simzar Hosseinzadeh, Yousef Fatahi, Masoud Soleimani, Fatemeh Atyabi, Rassoul Dinarvand

**Affiliations:** 1. Department of Pharmaceutics, Faculty of Pharmacy, Tehran University of Medical Sciences, Tehran, Iran; 2. Nanotechnology Research Center, Faculty of Pharmacy, Tehran University of Medical Sciences, Tehran, Iran; 3. Department of Polymer Engineering, Amirkabir University of Technology, Tehran, Iran; 4. Department of Stem Cell Biology, Stem Cell Technology Research Center, Tehran, Iran; 5. Faculty of Advanced Technologies in Medicine, Shahid Beheshti University of Medical Sciences, Tehran, Iran; 6. Department of Hematology and Blood Banking, Faculty of Medicine, Tarbiat Modaress University, Tehran, Iran

**Keywords:** Bioprinting, Cell differentiation, Extracellular matrix, Neurodegenerative diseases, Tissue engineering

## Abstract

Cellular transplantation, due to the low regenerative capacity of the Central Nervous System (CNS), is one of the promising strategies in the treatment of neurodegenerative diseases. The design and application of scaffolds mimicking the CNS extracellular matrix features (biochemical, bioelectrical, and biomechanical), which affect the cellular fate, are important to achieve proper efficiency in cell survival, proliferation, and differentiation as well as integration with the surrounding tissue. Different studies on natural materials demonstrated that hydrogels made from natural materials mimic the extracellular matrix and supply microenvironment for cell adhesion and proliferation. The design and development of cellular microstructures suitable for neural tissue engineering purposes require a comprehensive knowledge of neuroscience, cell biology, nanotechnology, polymers, mechanobiology, and biochemistry. In this review, an attempt was made to investigate this multidisciplinary field and its multifactorial effects on the CNS microenvironment. Many strategies have been used to simulate extrinsic cues, which can improve cellular behavior toward neural lineage. In this study, parallel and align, soft and injectable, conductive, and bioprinting scaffolds were reviewed which have indicated some successes in the field. Among different systems, three-Dimensional (3D) bioprinting is a powerful, highly modifiable, and highly precise strategy, which has a high architectural similarity to tissue structure and is able to construct controllable tissue models. 3D bioprinting scaffolds induce cell attachment, proliferation, and differentiation and promote the diffusion of nutrients. This method provides exceptional versatility in cell positioning that is very suitable for the complex Extracellular Matrix (ECM) of the nervous system.

## Introduction

The Central Nervous System (CNS) is a complex organ with specific restrictions, such as the limited capacity of the neuronal cells in proliferation and regeneration of damaged neurons in neurodegenerative disorders (Alzheimer's, Parkinson's, and Huntington disease, trauma, and stroke) ^1^. The Blood-Brain Barrier (BBB) is the main obstacle against crossing drug molecules and pharmacotherapy. Furthermore, complex neurobiology, lack of animal models for simulating the human brain, difficulty in achieving targeting effects, subjective clinical findings, high placebo responserates, the rarity of reliable biomarkers, and weak replicability of results even *in vitro* have forceddrug discovery to face with serious challenges ^2,3^. The most prominent element in neurodegenerative disorders arising from disease, stroke, and traumatic injuries, is the death of neurons ^1^.

On the other hand, the incapability of neurons in proliferation leads to disease progression over time, while the current treatments are only able to slow down the progression of neurodegenerative diseases ^4^. Historically, treatment success of CNS disorders has the lowest rate in the clinic among all therapeutic categories except for oncology and women's health ^2^. Lack of robustness in the preclinical findings, bias in the reporting of preclinical failures, and absence of robustness in the clinical trials are the main reasons for unsuccessful therapeutic approaches ^3^. One of the promising approaches in this area is the use of stem cells to repair damaged structures ^5^. There are two strategies for using cells; exogenous cell transplantation and endogenous cell stimulation ^6^. For effective cell transplantation, an ideal donor stem cell subtype, which matches with the pathophysiological requirements of individual disease, should be selected and appropriate host brain environment should be provided for improving donor cell survival. Moreover, neuroprotective and neurotrophic agents should be used to prevent further deterioration ^7^.

Although cell therapy has been presented as a promising option in neurodegenerative treatments, unsatisfactory performance is usually observed due to poor integration and cell survival, ineffectual lesion filling, and uncontrolled differentiation ^8^. Therefore, the engineering of a multifactorial scaffold containing a combination of cells, neurotrophic, and regulator agents is required to simulate neural stem cell niche microenvironment to improve cell survival, attachment, proliferation, differentiation, and migration ^8^.

Multifactorial scaffolds, which affect the nervous system *via* various mechanisms, have been more successful in the regeneration and recovery of CNS function. For example, the aligned conductive polypyrrole/poly (Lactic acid) (PPy/PLA) nanofibrous scaffold with bone marrow stromal cells instates nerve conduction by recovering the electrophysiological properties. This scaffold inhibited scar tissue formation, compensated for the lack of cells, and improved axonal myelination and regeneration in the lesion site ^9^. In another study, transplantation of Mesenchymal Stem Cells (MSCs) using rotary jet-spun porous PLA microfibers (Rough microstructure) to central nervous system injury, resulted in no inflammatory response, reduced the lesion area, and induced a 50% increase in C-X-C motif chemokine 12 (CXCL12) secretion by MSCs. CXCL12 is a more important factor in MSCs retention at the sites of injury ^10^.

In a study, Yang *et al* designed highly homogeneous and reproducible 3D-MnO_2_ nanoscaffolds by a vacuum filtration method from 2D-MnO2 nanosheets. They coated scaffolds with laminin and loaded N-[N-(3,5-Difluorophenacetyl)-L-alanyl]-S-phenylglycine t-butyl ester (DAPT). This platform increased beta-III tubulin expression, enhanced neuronal differentiation and neurite outgrowth in seeded human induced Pluripotent Stem Cell-Neural Stem Cell (hiPSC-NSC) by providing controlled chemical (Sustained-release neurogenic DAPT), physical (Scaffold structure), and biological [Laminin as Extracellular Matrix (ECM) component] properties ^11^.

In this paper, an attempt was made to briefly discuss different requirements, which play a role in natural neural tissue. A suitable scaffold with ideal features, as mentioned above, should be capable of supplying transplanted cells to be differentiated to desired cell type and finally integrated with microenvironment and other cells.

### Characteristic of CNS

***Anatomy of CNS:*** In general, the description of the anatomical nervous system is formed by two parts consisting of the central (includes the brain, spinal cord, and retina) and the peripheral nervous system.

Spinal cord and retina are distinct parts, but the brain is a more complex structure. The cerebrum, the diencephalon (the thalamus and the hypothalamus), the cerebellum, and the brainstem are four major regions of the brain, and among them, the cerebrum is the largest portion.

**Figure 1.**
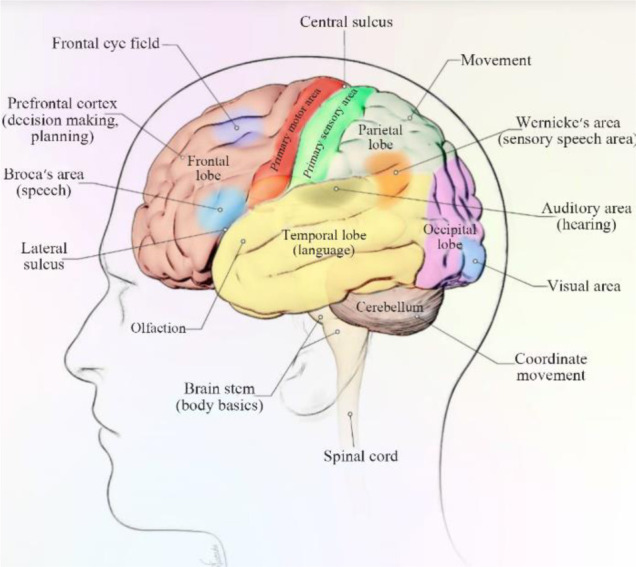
The lobes of the human cerebral cortex (lateral view) and some functional regions of the cerebrum.

The cerebrum is divided into two hemispheres. The cortex of cerebrum is arising from wrinkled gray matter which is responsible for higher functions of the brain. Folding of gray matter helps it to be placed in a small volume of the skull. Localization of function is an important property of the brain; it means that each region of the cerebral cortex or every other part is responsible for a specific function (Figure 1). Thus with damage to a part of the brain, the specific function of that part will be disrupted. For example, area 9 and 10, Dorsolateral anterior Prefrontal Cortex (DLPFC), is responsible for the motor organization, planning, and regulation. Dysexecutive syndrome is caused by damage to this area. This syndrome can affect executive memory, social judgment, and abstract thinking and intentionality^12^.

Diencephalon is another part of the brain, which is the connection between cortex and nervous system except for olfactory nerve. The brain stem is composed of three parts; the midbrain, pons, and medulla. The midbrain has the task of coordinating visual, auditory, and somatosensory perceptual spaces. Several vital functions are regulated by pons and medulla, such as heart and respiratory rate. The cerebellum balances the descending instruction from the cerebrum with sensory information from the periphery. Therefore, it acts as a small brain.

### Cellular characteristics of CNS

The cellular characteristics in CNS help for the comprehension of the neurochemical features and spatial structures of different cell types in communication to their surroundings. These cells are divided into two main types consisting of neurons and glial cells. Neurons are highly specialized and distinguished cells, which are the major component of CNS and the most important reason for brain complexity. Indeed, approximately 100 billion neurons are arranged next to each other that has created a very grand and complex network. In this network, each neuron is connected to 5000–10000 other ones. Thus, the rate of their communication and information transfer is incredibly quick, which at first sight is not dissimilar to an electrical on-off switch ^13^. Neurons can convey electrical signals through synaptic spaces. The produced wide network connection provides an immediate relay of information throughout the nervous system ^14^. Neurons structurally comprise of dendrites, cell body, and axon. For the transduction of signals, synapses must be formed.

Neuroglia cells have occupied a large mass of brain alongside neurons. While many works of literature and textbooks have declared glia outnumber neurons by as much as 10 to one, and some have gone a step further and say this ratio is 50 to 1 ^15^, it should be noted that this ratio is obtained due to counting the cells in some areas of the cortex and extending that to the entire brain while there are many diversities of neurons in different sections of the brain. Azevedo *et al* introduced a new, highly efficient method for counting brain cells in 2009 ^16^. According to their study, the ratio of glial cells to neurons in the overall human brain is 1 to 1. The proportion of glia to neurons may not seem so important, but some scientists believe the perception of which brain cells die or survive over the aging can induce development of new treatments for the neurological diseases which involve the loss of brain cells. Apart from all these interpretations, the number of glial cells, whether greater than or equal to neurons, has an important role in the development and health of nerves system throughout the lifetime. Although glial cells do not conduct the electrical impulses, and some scholars considered them as non-nerve cells, but their influence on the electrical behavior of neurons and their functional versatility is not negligible. Indeed, these cells result in neuronal survival, differentiation, neurite outgrowth, and synaptogenesis, trophic, and metabolic support of neurons, regulating the local concentrations of ions and neurotransmitters ^17^.

Glial cells in the Peripheral Nervous System (PNS) include Schwann Cells (SCs) and in the CNS include the oligodendrocytes, astrocytes, ependymal cells, choroid plexus, and microglia.

### Signaling of neurons

The communication language between the various cells of the body is carried out through electrical and chemical signals. In recent decades, a third factor, *i.e*., mechanical signals that are effective in cell-cell and cell-ECM communications, has been proven ^18^.

Chemical signals are lipids, proteins, or evengases secreted by cells that affect the neighboring or distant cells. Electrical signals are changes in the overall balance of negative and positive ions inside and outside a cell that transmit signals along the cellmembrane. Mechanical signals are changes in the different forces on the cell membrane.

### Biochemical signaling of neurons

The chemical signals are chemical molecules that are released from specific cells and affect the neighboring or distant cells. These signal molecules include amines, steroids, proteins, and small molecules, such as Adenosine Triphosphate (ATP), Deoxyribonucleic Acid (DNA), and Ribonucleic Acid (RNA). Therefore, the ligand (chemical molecule), and transmembrane receptors (Receiver) are required for chemical communication. In the neural network with the arrival of the action potential at the end of the axon, neurotransmitters are released from synaptic vesicle exocytosis, diffuse across the synaptic space, and dock with specific receptors on the other side of the synaptic space cell membrane. These receptors made the conversion in a post-synaptic cell by altering polarization ^19^.

A chemical signal is a path for the transmission of electrical signals in regions with slight distance between cells (synaptic space) which do not provide the possibility of transferring electrical signals. In addition, neural cytokines, Growth Factors (GFs), and neurotrophic factors are other chemical cues that bind to transmembrane receptors which affect cell fate. Brain-Derived Neurotrophic Factor (BDNF) is well known as a factor that promotes survival and neurogenesis through the tropomyosin receptor kinase b (TrkB) receptor ^20^.

Nerve Growth Factor (NGF) and Fibroblast Growth Factor-2 (FGF-2) are other factors that act through p75 receptor and FGF receptor 1, respectively ^21,22^. Furthermore, neurotransmitters can affect neurotrophic factors, for instance, α_2_-adrenergic agonist increases BDNF and Vascular Endothelial Growth Factor (VEGF) in local noradrenergic afferents ^23^ and serotonin can stimulate BDNF expression ^24^. In table 1, the major neurotransmitters and their site of actions are shown.

**Table 1.** Main neurotransmitters and their site of action

**Neurotransmitter**	**Type of action**	**Region of activity**	**Ref**
**Acetylcholine**	Excitatory	CNS and PNS - Acetylcholine induces mesenchymal stem cell migration - ACh increased the viability, but decreased the proliferation of embryonic stem cells and improved intestinal epithelial stem cell proliferation	[25–27]
**Norepinephrine**	Excitatory and inhibitory	CNS and PNS - It increased number of neurites, enhanced cell survival, while proliferation was inhibited	[28]
**Histamine**	Excitatory	CNS and PNS - Histamine induces neural stem cell proliferation and neuronal differentiation by activation of distinct histamine receptors	[29]
**Glutamate**	Excitatory	CNS and PNS - ASCs proliferation rate was significantly reduced in the absence of glutamine. - The specific activation of group I mGluRs (mGluR1 and mGluR5) increases the expression of leukemia inhibitory factor (LIF) and brain-derived neurotrophic factor (BDNF)	[30–32]
**Aspartate**	Excitatory	- Performs important roles related to nervous system development and hormone regulation - These data support the notion that D-Asp is involved in neuronal differentiation.	[33]
**Dopamine**	Excitatory and inhibitory	CNS and PNS - Dopamine-induced proliferation of adult neural precursor cells in the mammalian subventricular zone	[34]
**Nitric oxide (NO)**	Evokes the release of several neurotransmitters, including acetylcholine, catecholamines, and neuroactive amino acids	- nNOS-derived NO is a negative regulator of adult neurogenesis in physiological conditions. NO is primarily a direct cytostatic agent in many cell types, including neuroblasts; thus, the neurogenic action of NO in damaged brain is due to its indirect effect, most probably up-regulation of VEGF	[35–37]
**Serotonin**	Inhibitory	- Brain, spinal cord and PNS (Enterochromaffin-like cells in GI)	[38,39]
**Endorphins**	Inhibitory	- CNS (Hypothalamus, striatum, spinal cord, hippocampus) and PNS	
**GABA**	Inhibitory	Brain, spinal cord and PNS GABA has depolarizing activity in cerebrocortical neural precursors, controlling cell division, and contributing to neuronal migration and maturation - It has a role in improving and accelerating the differentiation and functional maturation of human stem cell-derived neurons	[40,41]
**Glycine**	Inhibitory	Brain and spinal cord - It modulates NSC proliferation and controls brain development.	[42,43]

### Electrical signaling of neurons

Chemical signals are slow and sometimes very slow messengers owing to the need to transmit chemical ligands through blood or other fluids. However, many times, there is a need for a quick spark and response. The electrical signals exactly act as a thunderbolt and transmit messages from one part of the cell membrane to another or, less commonly, to an adjacent cell. Occurance of electrical signals in neural cells as well as muscle cells is more important than other cell types ^44^.

Neurons are excitable cells that can procreate and response to electrical signals. When they are at rest state, without transmitting electrical signals, their potential inside charge is negative relative to the outside and is −70 *mV*. This membrane potential is necessary for electrical transmission. More specifically, the electrical signals are controlled by the concentration gradient of some ions and ion channels. Inside the cells, high amount of K^+^ and less Na^+^ relative to the extracellular fluid exists. Na^+^/K^+^ ATPase pumps on the cell membrane make these present fixed concentration gradients. On the other hand, K^+^ leak channels are continuously open, and the result of K^+^ leakage out in the cytosol becomes electrically negative. In addition, intracellular protein anions help to keep the cytosol negatively charged.

**Figure 2.**
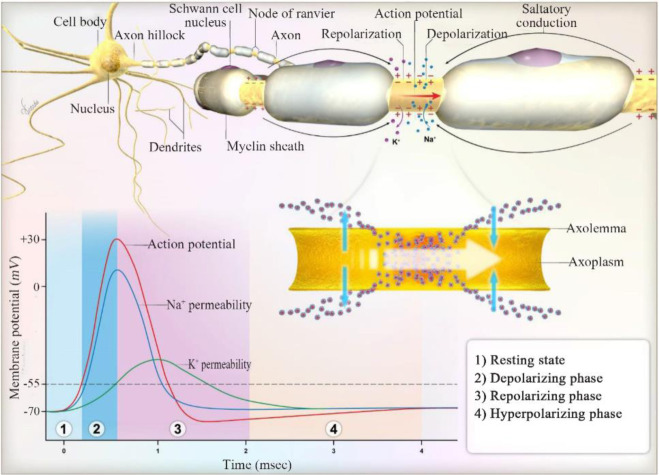
Electrical action potential in the nerve cells.

Each action potential in neurons occurs in four successive stages, as explained before; steady-state of cells is −70 *mV* approximately. The opening of sodium voltage-gated channels and incoming sodium ions, which describe the depolarization, elevate this resting membrane potential. When depolarization grows enough to bring the membrane potential up to the threshold (−55 *mV*), the action potential is activated. Thus, depolarization continues sharply until the membrane potential arrives at about +40 *mV*. There, sodium channels are inactivated but not closed. In this condition, cells will not respond to stimulations. Therefore, new stimulation occurs during absolute refractory. Repolarization of cell membrane takes place by the opening of K^+^ voltage-gated channels. K^+^ channels remain open for a long time that hyperpolarizes the membrane. Shortly before hyperpolarization, sodium channels come out of inactivation, but hyperpolarization needs strong stimulation to launch new action potential, and thus, the relative refractory period is produced. Na^+^/K^+^ ATPase pumps pump two potassium ions in and three sodium ions out. At this moment, cells become ready to be stimulated by another trigger. Therefore, an action potential is expanded for a brief duration. This temporary change in membrane potential is called an “electrical signal,” and it is the tool of electrical communication along the cell membrane and between cells (Figure 2).

Until recently, despite the advances in discovering the mechanism of signal transmission chemically, the mechanism of electrical transmission remained considerably unknown. It was imagined that electrical transmission is a rather simple, static, and rigid form of neuronal communication. Nevertheless, the current findings signify that electrical transmission is a dynamic and complex system. The electrical communication between coupled cells is modified with the involvement of some proteins in endocytosis or exocytosis and fast turnover of gap junction channels ^45^. These gap junctions are unique in their structure because they are electrogenic but are influenced by a neurotransmitter substance ^46^. Regulation of gap junctions occurs dynamically by the assembly, disassembly, or post-translational modifications and by different isoform composition. Gap junction channels can synchronize great neuronal ensembles at different frequency bands. In this way, they will sharpen the nervous activity as it happens in the cognitive process and learning ^47^.

Electrical transmission between two cells is established *via* clusters of intercellular channels that directly connect the internal space of two contiguous cells, which is called “gap junctions”. These channels are a type of two hemichannels called connexons, which are placed on the opposed membrane, and each connexon is composed of six connexin subunit proteins ^47^. Ions, metabolites, and small second messenger molecules such as cyclic Adenosine Monophosphate (cAMP) and Inositol Trisphosphate (IP3) can diffuse among two couple cells *via* connexons bi-directionally.

Different endogenous and exogenous mechanical forces such as stretch, tension, pressure, and stress are incessantly exerted on the plasma membrane of cells, which may activate mechanosensitive channels in the CNS. In contrast, the cells sense, transduce and respond to mechanical stimuli. Based on this, mechanical impulses can influence ion channel gating, vesicular transport, fluid homeostasis, cell adhesion, cell division, gene expression, cell migration, and morphogenesis ^48,49^.

The most important cell organelle, which plays amajor role in communication between cells and the environment, especially for scaffolds, is the plasma membrane. Plasma membranes are viscoelastic structures that are most sensitive to bending forces and at least sensitive to compression forces, which leads to exocytosis and recapturing the vesicles. On the other hand, mechanical forces cause membrane deformations that affect the activity of ion channels on millisecond timescales relevant to the neuronal activity ^34^. Donoghue *et al* have identified the appropriate topographical features of the substrate required for the design of a three-dimensional scaffold intended for transplantation in spinal cord injury. They used mouse embryonic spinal cord to produce myelin cultures. The results indicate that the myelination in the polymer substrate is delayed compared to the cultures plated on glass coverslips. In fact, the differentiation of oligodendrocytes is delayed compared to the glass coverslips and is not inhibited on Polycaprolactone (PCL) scaffolds. It is worth noting, however, that these results are not true for the nonporous PCL^50^.

Cytoskeletal elements such as actin, spectrin, microtubules, and neurofilaments can affect intrinsic viscoelastic properties of neuronal membranes by preparation of structural tension within a cell. Furthermore, these structures act as a three-dimensional array of force transducers, which can affect and regulate axonal growth cone dynamics, dendritic spine formation, plasticity ^51^, synapse formation and maturation ^52^. However, since our goal is to investigate the effect of external factors on the cell to select the appropriate scaffold, details of endogenous forces are better to be ignored. Nevertheless, it should be noted that these internal structures could provide molecular support for a host of ECM signaling mechanisms and influence the synaptic homeostasis and plasticity.

### Elasticity–softness of brain tissue

In the related studies of the brain mechanical properties and its storage and loss moduli calculations, most researchers assume that each sinus stress will lead to a sinus strain. Indeed, they consider the linear viscoelastic properties of the brain and the ease of adapting this linearity with real-time simulations. However, the brain is a structure with non-linear viscoelastic properties, and this behavior appears in the shear stress of more than 0.01 *MPa*. The shear stress depends on the rate and extent of deformation and on the whole time that the tissue is held in the deformed state ^53^. On the other hand, it should be noted that the mechanical properties of the CNS are not static and they dynamically change in the physiological processes, including tissue remodeling during wound healing, embryonic development, and pathological responses. Also, age, sex, and region of CNS are influences on stiffness. Researchers have shown in their studies on the brain of pig and rats that the immature brain of them could be approximately twice as stiff as that of adults. Therefore, a larger amount of force is required to deform the pediatric brain compared to the adult one ^54^. Söhl *et al* believed neurogenesis enhanced brain stiffness in adults ^47^. However, surveys indicate the doublecortin and polysialylated neural cell adhesion molecule expressing cells significantly decreased within the first year of life and several folds decreased from 20 to 100 years ^55^. Also, the remained neurogenesis occurs just in restricted areas of CNS such as dentate gyrus of the hippocampus ^56^. Therefore, it is more logical to assume that different experimental methods, technologies, and sample preparation protocols created these different data. Just like that, Weickenmeier *et al* authenticated that under situations with using the same method and protocols even for the same brain, they cannot identify a single and unique stiffness value to characterize the brain's stiffness ^57^. It has been approved that the stiffness of the brain is also different in some regions among males and females. The related research which was carried out by Campos-Cantón *et al* showed female occipital and temporal lobes are stiffer than males of the same age, respectively. According to the finding of this team, the stiffness of the brain in all groups was in range of 2.2–3 *kPa*, in different regions while this property changes in older populations as it becomes soft ^58^. Increasing myelin content results in the brain stiffness, thus immature and diseased brain, which has incomplete myelination, is softer than mature brain ^57^. On the other hand, the stiffness reported for the spinal cord tissue was in the range of 3–300 *kPa*, although different regions of the spinal cord possess different elasticity properties ^59,60^.

### ECM properties of brain tissue

Cells of each tissue are the main components that determine their appropriate functions, but they are not enough for this purpose. Indeed, cells are encircled by a complex matrix of various components, which is unique and specific to the same tissue ^61,62^. The CNS is not the exception from this principle, and ECM occupies considerable space of organ and forms a basal lamina surrounding the brain and blood vessels ^63^.

Based on this definition, initially, it was believed that ECM is an inactive matrix and its function is just limited to hold cells and tissues in place, but recent decades studies indicate that ECM is very effective in expansion, proliferation, migration, and differentiation of cells ^64,65^. The question that arises here is how these specific components are limited to a certain tissue. The studies that have been carried out so far may answer this question somewhat. For instance, the study of Sheppard *et al* focused on changing the distribution of ECM components during cerebrocortical development. According to their findings, fibronectin was first observed in the Ventricular Zone (VZ) -the most interior layer- and was abundant around the glial cells ^66^. During the developing process, differentiating neural cells migrated along the radial glial and under the influence of some ECM components such as laminin and fibronectin, toward higher levels and created the Pre-plate Zone (PZP) ^67^. It has been observed that fibronectin also moves along the migration of the neurons, so the VZ gradually lacked fibronectin ^66^. On the one hand, glia cells originally produced fibronectin in the early stage of developing, which was secreted by neurons eventually, and on the other hand, this may indicate the neurons’ need for the presence of fibronectin during differentiation.

Brain development is an on-going process, which is complete during pregnancy. This is an interesting point that the total number of neocortical neurons in newborn infants are as many as the one in adults while the total number of oligodendrocytes and astrocytes 3-fold increases in the first three years of life ^68^. The cause of lack of neuronal proliferation after birth is unknown. Moreover, it is not obvious why differentiation of glial cells into the neuron does not continue to increase the number of neurons. It is not clear whether ECM compositions or their ratio changed or not.

Of course, significant alterations occur in the composition and the content of ECM during development ^69^. However, unlike other cell types, the proliferation capacity of neurons is permanently blocked after their differentiation. They are typically existent in a quiescent state in the adult nervous system ^70^.

The ECM of the nervous system can be divided into three segments related to their compositions: the basement, a diffuse interstitial portion, and condensed structures, which surround the neural cells (but not all of them), which are named Perineuronal Net (PNN). PNN is the most specific part of CNS ECM, and its composition varies throughout development, as well as in different regions of the CNS ^71^. PNN plays a critical role in stabilizing the newly appointed neuronal connections, neuronal protection, limiting synaptic plasticity and neural regeneration, and modulation of the pathogenesis of various CNS diseases ^69,72^.

In the embryonic period, ECM occupies 40% of brain tissue, and in adults, this volume is reduced to 20% ^73^. Moreover, lamination of the brain is completed, and perhaps it can be said that neurons may require more widespread space for differentiation.

It may be essential to know whether the ECM affects the cells or the cells affect ECM. Certainly, there are bidirectional signals, which are evolved in response to multiple cues and induce various effects tailored to the needs. Over time, cells can remodel the matrix by changing the repertoire of matrix receptors in both the nature and quantity of constituent molecules ^74^. On the other hand, these dynamic modifications of the ECM can direct cell behavior ^74,75^.

### The components of ECM in CNS

ECM components are synthesized by cells and secreted into the extracellular environment to be used as substrates for cellular receptors, thus signaling events are started or induced across cell membranes. In other words, the physical structure and configuration of components produce a 3D environment that entraps signaling factors and therefore regulates the bioavailability of signals and effects cells behaviors ^75,76^.

Contrary to some imaginations that indicate migration of neurons during development is in the length of ECM fiber-like components orientation, the ECM in the CNS lacks the high proportion of fibrillar collagens and fibronectin that are typically found in other organs. However, these components accompany laminin, dystroglycan, and perlecan in the basement, which is a portion of ECM surrounding the blood vessels and endothelial cells. The basement has the most capability in regeneration among different compartments of CNS ECM ^77^. There are several types of laminin with various effects on the ECM of CNS. For instance, loss of laminin g1 prevents neurons from migrating towards the Marginal Zone (MZ) in the adult brain, and laminins a2/a4 are required for the formation of cell chains in the Rostral Migratory Stream (RMS) ^64^.

Chondroitin Sulfate Proteoglycans (CSPGs), Hyaluronic Acid (HA), and hyaluronan synthases, tenascins, and link proteins are luxuriant in diffuse interstitial and PNN ^53,78^. CSPGs possess more than 15 known iso-forms in the brain ^71^. Glucuronic acid and N-acetylgalactosamine are two disaccharide units repeated in Chondroitin Sulfates (CS) and covalently attach CS to the serine residues of a protein making CSPGs. The protein is the core of CSPGs and its length effects the biological activity of an individual proteoglycan. Besides that, the varying number of glycosaminoglycan (GAG) side chains and the sulfation patterns of the N-acetylgalactosamine and glucuronate disaccharide are effective in its biological activity. Chondroitin 6-O-sulfotransferase-1 (C6ST-1) and chondroitin 4-O-sulfo-transferase-1(C4ST-1) are responsible enzymes for sulfation. C6ST-1 is prevailing in developing the brain whereas C4ST-1 is dominant in the adult brain and inhibits cerebellar granular neurons growth ^71^. It may be thought the increment expression of C6ST-1 or reduced expression of C4ST-1 helps the growth of neurons and regeneration process. Nevertheless, studies show that overexpression of C6ST-1 caused impaired PNN formation and PV cell maturation, although underexpression of C6ST-1 causes poor regeneration in the CNS lesion ^79^.

In addition to the components mentioned above, chemotropic and trophic factors are also effective components of ECM. These factors, such as NGF, BDNF, and FGF, promote the expansion and differentiation of stem cells in CNS ^80^. Different ECM components and their main functions in CNS are collected in table 2.

**Table 2.** ECM components of CNS and main functions

**Classification**	**Components**	**Function**	**Ref**
**Adhesion molecules**			
	- Cadherin family - Integrin family - Connexin 43 - Fibronectin - Laminin	- Cadherin mediates cell-cell adhesion *via* homophilic interactions between the extracellular domains of cadherins on adjacent cells - Integrin mediates cell-ECM interactions *via* directly binding to ECM proteins such as laminin, collagen, and fibronectin	[81,82]
**Synaptic cell adhesion molecules**			
	- Cadherins - Ig-CAMs ^1^ - Neurexins - Neuroligins - Ephrins - Eph receptors	- These groups are not only involved in physical adhesion but also can control synapse formation, modify synaptic receptor function in an activity-dependent manner, and regulate dendritic spine morphology	[83,84]
**Proteoglycans**			
	- Heparan sulfate - Chondroitin sulfate (CS) - Dermatan sulfate - Keratin sulfate - Hyaluronan - Reelin - Tenascin family	- They participate in the regulation of brain development, maturation, normal brain function, and play key roles in neurodegenerative diseases	[67,85]
**Neurotrophic factors, and growth factors**			
	- NGF ^2^ - BDNF ^3^ - NT-3 ^4^ - NT-4/5 - CNTF ^5^ - GDNF ^6^ - Galanin - Sema3A	- They enhance the growth of the axons	[86,87]
**The thrombospondin type 1 repeat (TSR) superfamily**			
		- They regulate matrix organization, the guidance of cell and growth cone migration, and cell-cell interactions	[88,89]

1- Immunoglobulin-containing cell adhesion molecules,

2- Nerve growth factor,

3- Brain-derived neurotrophic factor,

4- Neurotrophin-3,

5- ciliary neurotrophic factor,

6- Glial cell line-derived neurotrophic factor.

### Scaffolds for the CNS tissue engineering

Restrictions on the proliferation of nerve cells in different CNS damages, as well as the formation of scar glial, lead to producing an inhibitory environment against cell expansion, migration, proliferation, and axonal extension ^90^. On the other hand, the shortcoming of traditional cell suspension transplantation, which is unable to provide appropriate mechanical and physical support for optimal differentiation of cells^91^, has made it necessary to employ different scaffolding. An important consideration in the design of scaffold for CNS is that it must be able to mimic the natural tissue and remain sufficiently intact and stable so that axons can elongate through it ^92^.

On the other hand, communication between cells, diffusion of oxygen and nutrients, and waste products require an interconnected channel to easily flow media around cells. When the designed scaffolds are more similar to ECM of CNS, it is easier to reach the target. The ECM is a particularly rich source of signals, a reservoir of GFs, acting as structural support and transducer of mechanical signals ^93^. ECM in neural tissue mainly affected neurite length, neuronal adhesion, and mechanotransduction, which is often associated with structural scaffolding ^94^. In this regard, some researchers have used 3D substrates to simulate the neural culture medium to the real micro-environment of the brain and have studied the neural networks and synaptic plasticity ^94^.

In 3D scaffold design, differentiation-inducing factors including GFs or ECM components can usually be immobilized by a linker, or encapsulated in the scaffold with an adjustable release manner ^95^. These bioactive molecules play critical roles in governing the cellular fate of the stem cells ^96^. 3D scaffolds can organize stem cells into a higher-ordered construct to achieve the neural tissue function. 3D neuronal networks, which are the zenith of perfection, can control the position and direction of neuritis outgrowth and closely mimic the actual CNS structure. As compared to 2D systems, NSCs in 3D systems extend longer neuritis and follow a random migration pattern, and present different electrophysiological properties ^91^.

**Figure 3.**
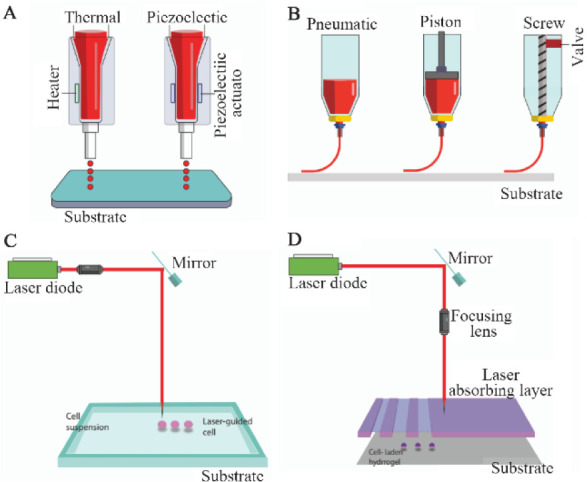
The structure and operation of available bioprinting methods: A) Inkjet printing method: In this method, air pressure pulses or mechanical pulses are used to eject the hydrogels/droplets. B) Microextrusion printing method: It uses the pneumatic, piston- and screw-based mechanisms to supply a continuous flow of bio-inks. C) Laser-guided direct cell printing method: This method influences the difference in refractive indices of cells, culture media to trap and assist them onto a receiving substrate. D) Laser-induced direct cell printing method: The vapor bubble is created by the laser and results in the removal of the hydrogel droplets from the absorbing layer.

Subsequently, different scaffolds designed to be used in the nervous system are introduced, among which aligned systems, conductive, injectable, and soft scaffolds are specifically designed for neural systems. Also, 3D printing with or without cells has recently opened an interesting window to design controllable scaffolds. The bioprinter is used to provide 3D architecture. In this method, after the bioink is prepared, the spatial patterning is done, and the scaffolds are made in three dimensions with very accurate geometry. Figure 3 gives an overview of this process. This new approach will be expanded. At the end of this article, the details of this method are given ^97^.

### Aligned systems for CNS tissue engineering

The transmission direction of the nerve signals and the integration of transplanted cells with host cells are affected by axonal orientation. Therefore, using substrates with the longitudinal orientation can simulate nerve conduits, promote the growth and orientation of regenerating axons, and play a critical role in direct cell migration. One of the most successful methods that can influence cell migration and orientations is using a magnetic field. Esmaeili *et al* illustrated applying an external electromagnetic field on MSCs results in cell alignment toward the identical direction. This alignment significantly increases the differentiation of MSCs to neural cells. They achieved differentiated neural cells just using an electromagnetic scaffold without chemical differentiation factors ^98^. In another study, Xia *et al* profited from magnetic fields to the orientation of cells containing superparamagnetic iron oxide nanoparticles. They magnetofected SCs by poly sialyltransferase functionalized superparamagnetic iron oxide nanoparticles. Applying the magnetic field significantly increases SCs migration into the astrocyte domain ^99^. Using magnetic fields to the orientation of the scaffold structure, which contains magnetic properties, is another method to help the preferential direction of cells ^100^. Design of grooves and multichannel texture as an aligned pattern and printing them on mold, producing microfibers, and 3D matrix fillers with longitudinally-oriented architectures are arising from the aligned scaffold idea ^101^. Yang *et al* used multichannel scaffold for differentiation of Activated Schwann Cells (ASCs) and MSCs for neural regeneration of spinal cord injury in rats. They have seen growth and branching of axons fibers through microchannels that can bridge two sides of the lesion together ^102^.

In summary, cell adhesion, cell migration, proliferation, and cell differentiation are affected by the cellular microenvironment. Investigating the relationship between cellular deformation and cellular microenvironment is a topic of interest to biology researchers. For example, Zhang *et al* have tested zigzag microgroove surfaces, which mimic the ECM of the tendon, and the effects of various ridge lengths, ridge angle, ridge widths and groove widths on MSCs were investigated ^103^.

Electrospinning is one of the most common means for the preparation of aligned nanofibers. The advantages of this method are simplicity, ability to integrate with large scale processing, accurate controllability, quick tunability, and economic importance ^104^. For example, Weightman *et al* used this method to prepare multicellular implantable scaffolds to repair nerve injury. They prepared fluorescent and non-fluorescent poly-L, D-lactic acid nanofibers *via* this method. Elongation and maturation of Oligodendrocyte Precursor Cells (OPCs) following culture with pre-seeded astrocytes on nanofiber-hydrogel constructs were shown ^105^.

However, specialized equipment requirements, the high voltage that limits using biological substances, conducting targets, and the limitation for the use of a variety of polymers are drawbacks of electrospinning^106,107^. Applying an electrical field on the polymeric solution by itself can induce a one-dimensional crystallization process to produce micron-sized fibers. These microfibers start to grow, from the edge of the electrodes, in the direction parallel to the electric fields, and move towards the center of the gap ^104^.

In addition, researchers have used various techniques for the incorporation of aligned appearance into scaffolds. For instance, it is recommended to use wire or needle or different polymers, that can be removed or dissolved after an engraving of the pattern ^108^.

### Conductive scaffolds for CNS tissue engineering

Neurons are electrically responsive cells, and electrical stimulation has been proven to have a beneficial effect on neuronal function and nerve regeneration ^109,110^. In this regard, electrically conductive scaffolds have been considered as an attractive approach for neural tissue engineering.

A variety of conductive biomaterials have been developed for use as tissue engineering scaffolds, which can be placed in three main classifications:

1. Conductive metallic based nanoparticles, such as gold, silver,
2. Carbon-based Nanoparticles (NPs) such as Carbon Nanotube (CNT) and graphene oxide nanoparticles,
3. Conductive polymers that are applicable in various types of scaffolds including pure conducting polymer films, conducting blends or composite films, conducting copolymer films. These materials could be provided as conducting nanofibers, conducting hydrogels, or 3D conducting composite ^111^.

These categories are usable as the conductive structure itself, either solely or in combination with an electric field. There are three methods for categorizing how these materials can be used for fabrication of scaffolds:

1. Accommodation of conductive material on the surface of the prepared scaffold, like what Baranes *et al* did to incorporate AuNPs on the electrospun fiber scaffolds by evaporation of gold ^112^. Their results illustrate that the axonal elongation of neurons, which were cultivated on the gold nanoparticle scaffolds, is dominant relative to forming complex branching trees.
2. Incorporation of conductive materials into the scaffold structure, for instance, in the study carried out by researchers such as Zhou *et al* who made a conductive scaffold using embedded CNT in PCL solution. They found out electrical stimulation of fabricated conductive scaffold enhanced PC-12 cell proliferation and neurite extension and promoted intracellular connections and cellular migrations ^113^.
3. Fabrication of conductive polymer-based scaffolds. Gu *et al* produced biodegradable and conductive biomimetic nanofiber from natural chitin and conductive Polyaniline (PANi) blended solutions. They observed excellent viability of hMSCs^114^.

Some researchers believe Conducting Polymers (CPs) exhibit great advantages for use as scaffolds in CNS tissue engineering ^115^. These materials exert electrical properties akin metals. Furthermore, they are biocompatible and capable of increasing cellular activities such as cell adhesion, proliferation, migration, and differentiation with or without electrical stimulations ^116,117^. Various conducting polymers, including PANi and Polypyrrole (PPy), have been investigated as conductive scaffolds for neural tissue engineering ^118^. Xie *et al* investigated the potential of conductive core-sheath nanofibers in neural tissue engineering. Electrical stimulation was found to further increase the maximum length of neurite compared to the control group without electrical stimulation ^119^. In another study, the 3D electroactive PPY/collagen fiber scaffolds were used to differentiate hMSC to neuronal cells. The results showed the upregulation of neural markers in the MSC following the external electrical stimulation ^120^. Although many studies have confirmed the compatibility of conductive polymers such as PPy, the existence of other studies have rejected the findings and directed researchers to contemplate further. Ferraz *et al* observed in their study on PPy-nanocellulose composite that although extensive and multiple rinsing can reduce the conductivity, it creates a nontoxic structure by removing impurities ^121^. Recently, Liu *et al* used functionalized CNT with Poly Ethylene Glycol (PEG) cross-linked with double bonds. The final carbon-nanotube-PEG-acrylate (CNTpega) material was embedded within oligo (poly(ethylene glycol) fumarate) (OPF) at different concentrations to form conductive hydrogels with modulable conductivities for spinal cord injury. The results showed that with increasing CNT content, cell density decreased. Also, the differentiation abilities of PC12 cells on these hydrogels were evaluated by culturing cells with culture media containing 50 *ng ml*^−1^ NGF. After induction of cell differentiation with NGF, the cells on pure oligo-PEG-fumarate still had the same morphology with a rounded shape. While on the conductive hydrogels embedded with CNTs, the cells all showed a higher number of cells, consistent with the proliferation test ^122^. Table 3 presents some examples of conductive scaffolds in neural tissue engineering and their applications.

**Table 3.** Conductive scaffolds in neural tissue engineering applications

**Polymer type**	**Cell type**	**Induced factor**	**Duration (day)**	**Characterization**	**Key finding**	**Ref.**
**PLA ^1^, SWNTs ^2^, MWCNTs ^3^**	Mouse ESC ^4^	Retinoic acid	7	ICC ^5^, RT-PCR ^6^	Increased conductivity after CNT addition, induction of neural differentiation of mESC	[123]
**PCL, PLA, PPy^7^**	Dorsal root ganglia	Electrical stimulation	6	SEM, Q Imaging	The neurite extension on uniaxially aligned nanofibers could be uniaxially aligned and enhanced by 1.82-fold on random fibers. The maximum length of neurites increased by 1.47 and1.83-fold on the aligned and random nanofibers, respectively	[119]
**PANI ^8^, poly (ɛ-caprolactone)/gelatin (PG)**	C17.2 (mouse neuronal stem cells)	Electrical stimulation		MTS ^9^, FTIR ^10^, XPS ^11^ spectrum	Electrical stimulation through conductive nanofibrous PANI/PG scaffolds enhanced neurite outgrowth and cell proliferation compared to the absence of electrical stimulation	[110]
**Cellulose acetate, MWCNTs**	SH-SY5Y neuroblastoma cell line	-	15	Two-point probe system, confocal microscopy, SEM	Conductive cellulose-derived scaffolds provided good cell attachment, growth, and differentiation	[124]
**Co_2_-MWCNTs**	HBMMSSCs ^12^	-	22	RT-PCR, ICC	Upregulation of neural growth factors increased neural differentiation of hBMMSC	[125]
**Collagen I, CNT ^13^**	HdpPSC^14^	-	6	ICC, Beta-1 integrin blocking experiments	It accelerated neural differentiation	[126]

1- Poly (Lactic acid),

2- Single walled carbon nanotube,

3- Multi walled carbon nanotube,

4- Mouse embryonic stem cell,

5- Immunocytochemistry,

6- Reverse transcription polymerase chain reaction,

7- Polypyrrole,

8- Polyaniline,

9 3-(4,5-dimethylthiazol-2-yl)-5-(3-carboxymethoxyphenyl)-2-(4-sulfophenyl)-2H-tetrazolium,

10- Fourier-transform infrared spectroscopy,

11- X-ray photoelectron spectroscopy,

12- Human bone marrow mesenchymal stem cells,

13- Carbon nanotube,

14- Human decidua parietalis stem cells.

### Soft materials for CNS tissue engineering

Soft materials such as hydrogels are biocompatible and have been widely applied as CNS tissue engineering scaffolds due to their soft tissue-like properties. They can regulate cell behavior and tissue formation by providing an ECM as a mimetic microenvironment ^127^. Furthermore, due to their intrinsic biological activity, natural hydrogels can create tailored signaling to cells without the need for GFs ^128^. Hydrogels are hydrophilic polymeric material and can be filled with water, which results in permeability to oxygen, nutrients, and water-soluble metabolites ^129^. They can be made from natural polymers, including collagen, gelatin, alginate, HA, agarose, and chitosan or synthetic polymers such as PEG, Polyacrylamide (PAA), and Polydimethylsiloxane (PDMS) ^130^.

Mosahebi *et al* have used alginate gels to transplant Schwann cells into a nerve guidance conduit ^131^. Alginate has also been applied to fill cavities following spinal cord injury ^132^. In another study, Tian *et al* have employed hyaluronic acid-poly-D-lysine based three-dimensional hydrogel to treat traumatic brain injury ^133^. Chitosan also has been found to support the growth of neurons and glia in the cell culture ^134^. Among the synthetic hydrogels, methacrylate-based hydrogels have the ability to provide mechanical properties similar to neural tissue. Poly (2-hydroxyethyl methacrylate) (pH-EMA) has been widely used for neural tissue engineering in spinal cord injury. It was shown that pHEMA sponges, which were implanted into the injured spinal cord, could facilitate regeneration of adult rat brainstem motor axons ^135^ (Table 4).

**Table 4.** Examples of soft scaffolds for CNS applications

**Hydrogel type**	**Young module**	**Target tissue**	**Cell source**	**Finding**	**Ref**
**PA/matrigel**	E ~1.5 *kPa* soft	Brain	- Oligodendrocyte precursors (OPC) - Schwann cells (SC)	No significant differences in cell attachment, viability and/or proliferation were observed between the soft and rigid matrices, although it was observed that for E <1 *kPa*, OPC attachment and survival was not optimal	[136]
**PA/matrigel**	E ~30.0 *kPa* rigid	Spinal cord	- Oligodendrocyte precursors (OPC) - Schwann cells (SC)		
**Agarose, PEG–GelMa 3D printed scaffold**	260–300 *kPa*	Spinal cord	- NPCs	Injured host axons regenerate into 3D biomimetic scaffolds. The synapse onto NPCs are implanted into the device and that implanted NPCs, in turn, extend axons out of the scaffold and into the host spinal cord below the injury to restore synaptic transmission which significantly improves functional outcomes	[137]
**Matrigel 100%**	896±265 *Pa*				
**Matrigel 50%**	16±9 *Pa*				
**Matrigel 25%**	5±2 *Pa*	Brain, spinal cord	- ESCs	ESCs within 3D matrigel scaffolds and on collagen-1 coated 2D substrates were significantly differentiated to neurons with robust neurite outgrowth	
**Collagenat pH=9**	1071±321 *Pa*				
**Collagenat pH=7.4**	511±142 *Pa*			3D collagen-1 scaffolds enhanced significant motor neuron formation, while 3D matrigel stimulated dopaminergic neuron differentiation	[138]
**Collagenat pH=5.5**	326±78 *Pa*				
**HA at 5 *mg/ml***	90±27 *Pa*				
**HA at 2 *mg/ml***	22±8 *Pa*				
**HA at 1 *mg/ml***	1.2±0.3 *Pa*				
**1% alginate hydrogels with RGD peptide**	1.17±0.48			Living cells decrease with the alginate concentration	
**1.5% alginate hydrogels with RGD peptide**	2.62±0.77	Peripheral nervous system	- Schwann cells	This result illustrates that the cell proliferation would be preferred on the softer substrates	[139]
**2% alginate hydrogels with RGD peptide**	9.54±1.93			Lowest cell viability was observed in 1.5% alginate without RGD peptide	
**2.5% alginate hydrogels with RGD peptide**	12.53±2.57				
**3.75% (w/v) PVA cross-linked with 10 *kGy* γ ray**	4.31±0.28				
**3.75% (w/v) PVA cross-linked with 20 *kGy* γ ray**	6.81±0.06				
**3.75% (w/v) PVA cross-linked with 40 *kGy* γ ray**	12.3±2.82	CNS	- Neural stem/progenitor cells (NSPCs)	Generation of NSPC clusters similar to those in neurosphere cultures was best achieved by 3.75% (*w/v*) PVA gel irradiated at 10 *kGy*	
**7.5% (*w/v*) PVA cross-linked with 10 *kGy* γ ray**	7.32±0.91			In this condition, the cells were maintained in an undifferentiated state	[140]
**7.5% (*w/v*) PVA cross-linked with 20 *kGy* γ ray**	7.63±0.55				
**7.5% (w/v) PVA cross-linked with 40 *kGy* γ ray**	20.0±0.50				

### Injectable systems

The cystic cavity that causes injuries in the brain or spinal cord is a major obstacle for tissue repair in CNS. Injectable scaffolds have provided a promising approach for nervous system tissue regeneration. Unlike a pre-formed scaffold that possesses a certain shape prior to its application, injectable scaffolds are injected into the defect area and then form the shape *in situ*. This feature allows for site-specific delivery of solidifiable precursor scaffold and cell mixture into the cavities and irregularly shaped defects in a less invasive way than implantation. There are two basic forms of injectable scaffolds, including hydrogels and microspheres. Unique features of microspheres, small size, and large specific surface area make them a suitable cell carrier for tissue engineering. Hydrogels are the most widely studied injectable scaffolds in the field of tissue repair. It has been shown that shear-thinning injectable hydrogel was potentially used as a filler of Nerve Guidance Channels (NGCs) and subsequently caused peripheral nerve tissue regeneration ^141^. Various composites based on injectable hydrogels are also frequently used in tissue engineering. For instance, in an interesting study by Johnson *et al*, magnetic PLLA-SPION aligned electrospunnanofibers were successfully prepared, and then they were cut and rolled into conduits. By this trick and using the collagen solution, they benefited from the advantages of the injectable scaffold, while the magnetic field was used for *in situ* realignments of the nanofibers at the end of injection ^142^.

Various natural materials such as collagen, gelatin, chitosan, alginate, hyaluronic acid, fibrinogen, and synthetic polymers such as PEG, Poly (a-hydroxy esters), and poly (N-isopropyl acrylamide) (PiPA) were used as injectable systems. Furthermore, in recent years, self-assembled peptides have been considered as a new class of injectable scaffolds ^143^. Biomaterial scaffolds composed of purified natural polymers are biocompatible systems, that have desirable features for tissue engineering application such as architecture, stiffness, porosity, and precisely controllable degradation rate ^144^. Natural polymers present specific molecules for cell adhesion ^145^.

Overall, *in situ* gelling was formed based on several main mechanisms including chemical crosslinking [Photo-cross linkable (UV or gamma)]^146^, physical crosslinking including pH-responsive, thermally sensitive, peptide crosslinking ^147^ and enzymatical crosslinking ^148^. By suitable selection of guanosine 5'-diphos-phate as a chemical cross-linker, Mekhail *et al* could fabricate a rapidly-gelling chitosan sponge that had the most proper features including high porosity with interconnected pores, rapid gelation, cytocompatibility, modulus of elasticity resembling that of soft tissue. They succeeded in the differentiation of oligodendrocyte progenitor cells in 12 days and introduced an injectable sponge as a promising therapeutic modality that can be used to enhance remyelination^149^.

### Three-dimension (3D) printing

3D bioprinting is a bottom-up tissue fabrication technique, which is usually constructed from hydrogels that prints living structures layer-by-layer simultaneously along with cells or without cells and after fabrication, the print can be seeded with cells. In the first case, 3D bioprinting allows printing the cells directly onto the scaffold for optimal localization ^137^. For successful fabrication of scaffolds through 3D bioprinting, the specific organization of functional and supporting cell types, the composition of the extracellular matrix, communications between cells and microenvironment, and different effects that influence the fate of cells are main factors that must be understood carefully.

Neural tissues are not homogeneous, rather they contain different types of cells including various types of neurons, glial cells such as oligodendrocytes, microglia, astrocytes in the CNS ^150^, satellite and Schwann cells in the PNS ^151^, which are arranged with a high order spatial localization. On the other hand, the ECM of the nervous system is very complex with different biological, electrical, and mechanical forces ^152^. It seems that perfection of scaffold designing for tissue engineering is manifested in 3D printing of nervous tissue, where comprehensive knowledge is needed to embrace all aspects of the desired tissue.

There are currently three techniques of inkjet-based, laser or photo-assisted bioprinting, and microextrusion^153^. The selection of appropriate materials, which can provide specific features according to the special goal, is very important. Cytocompatibility is the first item that must be followed. In addition, cell adhesion, especially with low adhesion properties of neural cells is a Achilles heel for continuing the tissue engineering process. Printability is another important parameter, which should be considered. Viscoelastic property of selected materials is an important factor affecting the printability of materials before, during, and after the print ^97^.

Bioprinting is able to construct tissue models with uniform spacing and to provide exceptional versatility in cell positioning ^154,155^. Moreover, bioprinting can control the porosity of the scaffold and introduce interconnected channels. In addition, using 3D printed fiber meshes promotes cell growth, cell attachment, and diffusion of nutrients ^156^. 3D bioprinting has been known as a precisely controllable strategy for accurate fabrication of artificial biomimetic structures ^157^. However, this strategy, like the others, has some limitations. As discussed, the selection of optimal biomaterials is a critical factor in the successful use of bioprinting scaffold clinically. Many of these biomaterials are biologically too active that cause unwanted cellular interactions and premature or undesired stem cell differentiation. In addition, the mechanochemical structure of these materials is often different from optimal tissue constructs. Eliminatingthese limitations is a time-consuming process ^156^.

Koffler *et al* have fabricated 3D biomimetic scaffolds from PEG-gelatin methacrylate (PEG-GelMA) using microscale continuous projection printing method (*μCPP*) and then directly loaded them with NPCs. They observed injured host axons regenerate into 3D biomimetic scaffolds and produce synapse onto implanted NPCs. Implanted NPCs extend axons out of the scaffold and into the host spinal cord in the injured site ^137^.

It has been shown that using iPSC-derived spinal Neuronal Progenitor Cells (sNPCs) can adapt the homology of spinal host tissue so that they could be autologous to avoid complications with immune suppression. The 3D bioprinted living platform incorporating iPSC derived sNPCs, and OPCs can be precisely positioned within a neurocompatible scaffold *via* a one-pot printing process. By the 3D printing method, one can place multiple specific neural progenitor cell types in channels at a resolution of ≈200 *μm* and also control cell position and the direction of axon growth within the scaffold.

In all types of neurons, intracellular calcium signaling controls key cellular functions in neurocompatible 3D alginate-based scaffolds ^158^.

## Conclusion

Millions of people in the world suffer from an irreversible disability due to neurological diseases or damage to the nervous system; conventional treatments cannot completely cure these disorders.

In this review, CNS anatomy was first described and then the causes of low CNS regeneration were investigated. In summary, limitation in the proliferation of neural cells, restricted neural stem cells, limited areas, presence of some ECM inhibitory elements such as myelin and myelin-associated molecules, and various physical barriers due to glial scar are the causes of low capability of CNS regeneration. In recent years, tissue engineering has been applied to nerve regeneration in the nervous system in cases when nerve grafts are ineffective. Transplantation of cells to replace injured cells and to provide micro environment mimicking tissue structures containing neurotrophic factors results in induction of regeneration. Achieving optimum scaffold requires recognition of the structure and function of the nervous system as well as the composition and function of the ECM components. In other words, if the designed scaffolds are similar to ECM of demanded tissue, it will be easier to communicate between cells, transport nutrients, and remove waste products since the ECM in neural tissue affects key parameters that are associated with structural scaffolding, such as neurite length, neuronal adhesion, and mechanotransduction. Subsequently, various scaffolds were designed for use in the nervous system.

In this article, a collection of different factors involved in the selection and engineering of the ideal scaffold were explained. For this purpose, different scaffold systems used in CNS tissue engineering were explained *i.e*., aligned systems, conductive scaffolds, soft materials, injectable systems, and three dimensional printing. The longitudinal orientation of substrates that occur in the aligned systems can mimic nerve conduits, raise the growth and orientation of regenerating axons, and promote direct cell migration. It is obvious that neurons are electrically responsive cells. Thus, using electrically conductive scaffolds is an attractive approach for neural tissue engineering, forasmuch as the electrical stimulation has a beneficial effect on neuronal function and nerve regeneration. Soft materials can adjust cell behavior and tissue formation by providing an ECM as a mimetic microenvironment. Biocompatible hydrogels and other soft materials used in CNS tissue engineering have intrinsic biological activity since they create tailored signaling to cells without the need for GFs and due to the presence of water, they facilitate water-soluble metabolism. Injectable scaffolds offer a promising approach for nervous system tissue regeneration because they are injected into the defect area and then form the shape *in situ*. When there is a significant obstacle for tissue repair in CNS by the pre-formed scaffold, which has a specific shape prior to its application, this unique feature makes restoration possible. With the site-specific delivery of solidifiable precursor scaffold and cell mixture into the irregularly shaped cavities, treatments are made in a less invasive way than implantation. 3D bioprinting is a powerful, highly precise strategy to construct tissue models with uniform spacing, interconnected channels, controlled porosity which promotes cell growth/attachment and diffusion of nutrients. This method provides exceptional versatility in cell positioning that is very suitable for the complex ECM of the nervous system, although the optimization of parameters affecting this process is time-consuming. Also, different devices used in each system were expressed and compared with each other. Each method has its advantages and disadvantages and depending on its purpose, the user can adopt the appropriate method. Since the cellular microenvironment affects cell adhesion, cell migration, proliferation, and cell differentiation, choosing the proper system is an essential requirement.
